# *CFH* Y402H and *ARMS2* A69S Polymorphisms and Oral Supplementation with Docosahexaenoic Acid in Neovascular Age-Related Macular Degeneration Patients: The NAT2 Study

**DOI:** 10.1371/journal.pone.0130816

**Published:** 2015-07-01

**Authors:** Bénédicte M. J. Merle, Florence Richard, Pascale Benlian, Nathalie Puche, Cécile Delcourt, Eric H. Souied

**Affiliations:** 1 Ophthalmology Department, Hôpital Intercommunal de Créteil, University Paris Est Créteil, Créteil, France; 2 Université de Lille Nord de France, INSERM774 Institut Pasteur de Lille, Lille, France; 3 Université de Lille Nord de France, Lille, France; 4 CHRU de Lille, Lille, France; 5 APHP - Saint-Antoine Hospital, Endocrinology and Metabolic Disease Department, F75012 Paris, France; 6 Lille 2 University, School of Medicine, Department of Biochemistry and Molecular Biology, F-59045 Lille, France; 7 INSERM, Centre INSERM U897-Epidemiologie-Biostatistique, F-33000 Bordeaux, France; 8 Univ. Bordeaux, ISPED, F-33000 Bordeaux, France; International University of Health and Welfare, JAPAN

## Abstract

**Purpose:**

Genetic susceptibility could be modified by environmental factors and may also influence differential responses to treatments for age-related macular degeneration (AMD). We investigated whether genotype could influence response to docosahexaenoic acid (DHA)-supplementation in the occurrence of choroidal new vessels (CNV).

**Methods:**

The Nutritional AMD Treatment 2 (NAT2) study was a randomized, placebo-controlled, double-blind, parallel, comparative study, including 250 patients aged 55 to 85 years with early lesions of age-related maculopathy, visual acuity better than 0.4 Logarithm of Minimum Angle of Resolution units in the study eye and neovascular AMD in the fellow eye. Patients were randomized at baseline to receive either 3 daily fish-oil capsules, each containing 280 mg DHA, 90 mg EPA and 2 mg Vitamin E, or placebo.

**Results:**

Patients carrying the risk allele (C) for *CFH* Y402H had no statistically significant increased risk for developing CNV in the study eye (Hazard Ratio (HR)=0.97; 95% Confidence Interval (CI): 0.54-1.76 for heterozygous and HR=1.29; 95%CI: 0.69-2.40 for homozygous). Patients carrying the risk allele (T) for *ARMS2* A69S had no statistically significant increased risk for developing CNV in the study eye (HR=1.68; 95%CI: 0.91-3.12) for heterozygous and HR=1.78; 95%CI: 0.90-3.52 for homozygous). A significant interaction was observed between *CFH* Y402H and DHA-supplementation (p=0.01). We showed a protective effect of DHA-supplementation among homozygous non-risk patients. Among these patients, occurrence of CNV was 38.2% in placebo group versus 16.7% in DHA group (p=0.008).

**Conclusions:**

These results suggest that a genetic predisposition to AMD conferred by the *CFH* Y402H variant limits the benefit provided by DHA supplementation.

**Trial Registration:**

ISRCTN registry 98246501

## Introduction

Age-related macular degeneration (AMD) is the leading cause of blindness in industrialized countries [1]. It is comprised by two advanced forms, neovascular and atrophic AMD, both associated with severe visual impairment and generally preceded by early retinal abnormalities (drusen, pigmentary abnormalities). AMD is a multifactorial disorder involving genetic and environmental factors [2]. This disease has a strong genetic component and several genes have been identified as associated with AMD [2]. Complement factor H (*CFH*) [3–7] and *ARMS2* [8, 9] have been identified as the most important susceptibility factors. Subjects carrying the risk allele for *CFH* (C) have an increased risk for late forms of AMD up to 5 times and up to 8 to 10 times for *ARMS2* (T). Odds ratios increase with the number of risk alleles, and homozygous risk alleles have a stronger genetic risk. Others genes implicated in the complement pathway (complement factors *B*, *C2* and *C3*) [10–12], or in lipid metabolism (*ApoE* and *LIPC*) [13–15] have been identified as associated with AMD.

The influence of environmental factors such as smoking and diet have also been highlighted by epidemiological studies [16–20]. The Age-Related Eye Disease Study (AREDS) showed that a combination of antioxidants and zinc reduced the risk of progression to late AMD by 25% [21, 22]. Omega-3 polyunsaturated fatty acids (omega-3 PUFAs), particularly eicosapentaenoic acid (EPA) and docosahexaenoic acid (DHA), have anti-inflammatory, anti-apoptotic and anti-angiogenic properties. Even though AREDS2 [22] did not show any beneficial effect of omega-3 supplementation on AMD progression, observational studies are consistent and show that an increased dietary intake of these fatty acids is associated with a decreased risk for AMD [2, 16, 23–27]. Unmeasured confounders in observational studies might explain these contradictory results [28].

Genetic susceptibility could be modified by environmental factors and together these factors are highly predictive of AMD onset and progression [23, 29–33]. Genetic variations may also influence differential responses to AMD treatments. Few studies have investigated the interaction between nutrients and genetic risk, and their results have been inconsistent. With regard to the *CFH* genotype, some studies found an inverse association between AMD and antioxidants or zinc supplementation [31, 33], and AMD and dietary omega-3 PUFAs [23] among subjects carrying non-risk allele (T). In contrast, other studies [29, 32] have shown an inverse association for dietary omega-3 PUFAs or fish consumption for subjects carrying the risk allele for *CFH* (C). Analysis of interactions between *ARMS2* and omega-3 PUFA consumption revealed an inverse association for dietary omega-3 PUFAs among subjects carrying the risk allele for *ARMS2* (T) [23, 32].

The Nutritional AMD Treatment 2 (NAT2) study is a double-blind, prospective and randomized trial in patients with early AMD in the study eye and neovascular AMD in the fellow eye, receiving oral DHA or placebo over 3 years [34]. In patients with unilateral neovascular AMD, 3 years of oral DHA-enriched supplementation had the same effect as placebo on choroidal neovascularization (CNV) incidence in the study eye. However, red blood cells membranes fatty acid measurements revealed that CNV incidence was significantly reduced in DHA-supplemented patients showing a steadily high EPA+DHA index over 3 years. This finding suggested a preventive effect of high dosages of DHA. Due to the strong genetic component of occurrence of CNV and bilateralism in AMD, however, we hypothesized that the preventive effect of AMD might be influenced by the patient’s genetic background [35].

In the NAT2 study [34], we therefore investigated whether genotype could influence response to DHA-supplementation in the occurrence of CNV when fellow eye is affected with exudative AMD. We focused on two major AMD variants: *CFH* Y402H (rs1061170) and *ARMS2* A69S (rs10490924).

## Methods

Study procedures for the NAT2 study have been reported previously [34]. The authors confirm that all ongoing and related trials for this drug/intervention are registered. The study was reviewed and approved on July 18, 2003 by the relevant institutional review board (IRB), Comité de Protection des Personnes (CPP), Paris-Ile de France 5, Paris, France (S1 File). The final informed consent form was approved on October 20, 2003. It was conducted in compliance with local regulations and approved by the national advisory commission on databases computing personal information (Commission Nationale Informatique et Libertés). It complied with International Conference on Harmonization, Good Clinical Practice (ICH GCP) guidelines and the Declaration of Helsinki (1975, revised in 2000).

The study was declared to the International Standard Randomized Controlled Trial Number Register and was allocated ISRCTN98246501 registration number. Participants gave written consent for the participation in the study. Registering of the study was performed after first visit for the first patient but before last visit for the last patient. The delay for registering the study was due to poor experience in registering by the investigators at this time. At the time of the study design and launching, in 2003, although it was mandatory to have IRB (CPP) approval before starting any enrollment of the first patient in a randomized trial, and contrary to drug or surgical intervention trials, it was not mandatory to register nutritional/dietary intervention trials. When it became an obligation, since the NAT2 study is a randomized nutritional intervention relying on the use of natural products (omega-3 fatty acids in the form of triglycerides), the trial was then registered according to current regulations and recommendations, and registration occurred after the first patient was enrolled.

### Design

The NAT2 study was a double-blind, prospective, randomized, parallel, comparative trial in patients with early AMD in the study eye and neovascular AMD in the fellow eye, receiving oral DHA or placebo over 3 years. The assessment of incident CNV in the study eye was the primary efficacy endpoint.

### Study participants

Patients were enrolled prospectively from December 2003 to October 2005 in a single center at the Department of Ophthalmology, Hôpital Intercommunal de Créteil, Créteil, France. The last visit of first patient was December 2006 and last visit of last patient was in October 2008. Patients were examined at baseline (visit 1), 6 months (visit 2), 1 year (visit 3), 2 years (visit 4), and 3 years (visit 5).

Eligible patients were affected with early AMD (any drusen or reticular pseudodrusen with or without pigmentary changes) in the study eye and neovascular AMD in the fellow eye. The study eye was not affected by CNV at entry. Neovascular AMD was defined on the basis of fundus color pictures and fluorescein angiography examination. Inclusion criteria were as follows: (1) between 55 and 85 years old, (2) signed informed consent, (3) visual acuity better than +0.4 logarithm of minimum angle of resolution units in the study period [34]. The main exclusion criteria were: (1) CNV in both eyes or no CNV in either eye, (2) wide central subfoveal atrophy of the study eye and (3) progressive ocular diseases (severe glaucoma or other severe retinopathy) [34].

Patients were randomized at baseline (enrollment into NAT2) to receive either 3 daily fish-oil capsules, each containing 280 mg DHA, 90 mg EPA and 2 mg Vitamin E (Reti-Nat, provided by Bausch & Lomb), or placebo (602 mg olive oil) [34].

### Eye examination

Patients were examined at baseline, 6 months, 1 year, 2 years, and 3 years. Clinical and ophthalmologic examinations of potentially eligible patients were checked against inclusion/exclusion criteria at baseline. Recorded data included demographic information, relevant ocular and medical history, and concomitant treatment. The following examinations were performed at each visit: (1) best-corrected visual acuity, (2) slit lamp examination, (3) fundus photography, and (4) fluorescein angiography (FA).

FA was performed to screen for the presence of CNV at each planned visit, and in patients experiencing visual symptoms at any time during the study. FA was performed to detect the presence and specify the location (extrafoveal, juxtafoveal, or subfoveal) and type of CNV (classic or occult). Indocyanine green (ICG) angiography was performed to confirm the diagnosis in patients with suspected CNV.

### Laboratory testing

Biological samples were collected at baseline prior to any supplementation. These samples included serum lipids and lipoproteins (HDL- and LDL-cholesterol and triglycerides) and genetic polymorphisms validated as genetic markers of exudative AMD (*CFH* Y402H rs1061170 and *ARM2/HTRA1* A69S rs10490924).

Overnight fasting blood samples were delivered to a single clinical chemistry laboratory (Hôpital Saint Antoine, Paris) within 5 hours and were processed immediately. Serum total, HDL- and LDL-cholesterol and triglycerides were measured by enzymatic colorimetric and electrophoretic methods as previously described [36]. Genomic DNA was extracted from 10mL blood leukocytes as previously described in AMD patients [37] and using the Illustra kit according to the manufacturer’s protocol (GE Healthcare) in controls. Genotyping of *CFH* Y402H rs1061170 and *ARM2/HTRA1* A69S rs10490924 was performed by quantitative polymerase chain reaction allelic discrimination using reagents and conditions from Custom Taqman Single-Nucleotide Polymorphism Genotyping Assays (Applera, Corp, France), using ABI 7900HT (Applied Biosystems). Quality control of genotyping by Sanger sequencing and bioinformatics analysis were performed as described [37].

### Covariates

Socio-demographic factors were collected through face-to-face, standardized interviews at the same time as the baseline eye examination. These factors included age, gender, body mass index (BMI) [weight (kg)/height^2^ (m^2^)] and smoking status (never smoker or ever smoker).

### Statistical analyses

Comparisons between the DHA and placebo groups were performed using Pearson Chi^2^ for qualitative variables (gender, smoking, genetic polymorphisms and occurrence of CNV) and Student’s *t* test for quantitative variables (age, BMI and plasma lipids). Comparison between subjects who developed CNV and subjects who did not develop CNV were performed using Pearson Chi^2^ for gender and Student’s *t* test for age. Logistic regression was used to adjust these comparisons for age, gender and DHA treatment for smoking, BMI and plasma lipids. Variations of EPA+DHA in serum and in red blood cells membranes (RBCM) according to genotypes were evaluated using ANOVA.

Cox proportional hazards models were used to estimate hazards ratios (HRs) and 95% confidence intervals (CIs) for occurrence of CNV. Model 1 was adjusted for age, gender and DHA treatment. Model 2 was adjusted for the variables listed above plus BMI, smoking status, HDL-cholesterol, triglycerides, and *CFH* Y402H and *ARMS2* A69S polymorphisms. Global p-values were obtained assessing each genotype using the number of risk allele (0, 1 or 2). Additional models were constructed to determine interactions between genetic variants and DHA treatment. Using a multiplicative model, interaction terms for the number of risk alleles and DHA treatment were assessed separately for each variant. A significant interaction between DHA treatment and the variant indicates that the effect of the treatment differs by genotype.

For analyses presented in tables 1, 2, 3 and 5, differences were considered significant at p<0.05. For analyses presented in Table 4, the Bonferroni correction for multiple testing was applied. Two interactions were evaluated and three tests were performed for each variant. The p value for significance is therefore p<0.025 for interactions and p<0.017 for the interaction with the genotypes of the respective variants. All statistical analyses were performed using SAS version 9.2 (SAS Institute Inc., Cary, NC).

**Table 1:** Baseline demographic, behavioral and genetic risk factors for age-related macular degeneration according to treatment group.

	Treatment		
Variables	DHA n = 127	PLACEBO n = 123	p-value^a^
**Gender**, n (%)			0.08
**Men**	39 (30.7)	51 (41.5)	
**Women**	88 (69.3)	72 (58.5)	
**Age**, mean (SD), years	74.0 (6.5)	73.0 (6.9)	0.28
**Smoking**			0.19
**Ever smoker**	48 (38.1)	57 (46.3)	
**Never smoker**	79 (61.9)	66 (53.7)	
**Body mass index,** mean (SD), kg/m^2^	25.6 (4.1)	25.9 (4.0)	0.47
**Plasma lipids**, median (5th-95th percentiles) or mean (SD), mmol/L			
**Triglycerides**	0.97 (0.49–2.31)	1.00 (0.46–2.07)	0.23
**HDL-Cholesterol**	1.82 (0.53)	1.75 (0.55)	0.31
**LDL-Cholesterol**	3.85 (1.08)	3.75 (0.87)	0.42
**Genetic polymorphisms**, n (%)			
***CFH* Y402H**			0.16
**TT**	24 (18.9)	34 (27.6)	
**CT**	65 (51.2)	50 (40.7)	
**CC**	38 (29.9)	39 (31.7)	
***ARMS2* A69S**			0.97
**GG**	34 (26.8)	32 (26.0)	
**GT**	60 (47.2)	60 (48.8)	
**TT**	33 (26.0)	31 (25.2)	
**CNV occurrence**, n (%)			0.80
**Yes**	38 (29.9)	35 (28.5)	
**No**	89 (70.1)	88 (71.5)	

NAT2 Study, n = 250, 2003–2005.

CNV: choroidal neovessels; DHA: docosahexaenoic acid; HDL: high density lipoprotein; LDL: low density lipoprotein; SD: standard deviation.

^a^p for Student’s t test or Pearson Chi^2^ test.

**Table 2:** Associations between baseline demographic and behavioral characteristics and occurrence of CNV.

	Occurrence of CNV		
Variables	No n = 177	Yes n = 73	p-value^a^
**Gender**, n (%)			0.21
**Men**	68 (38.4)	22 (30.1)	
**Women**	109 (61.6)	51 (69.9)	
**Age**, mean (SD), years	73.4 (6.8)	73.9 (6.5)	0.47
**Smoking**			
**Ever smoker**	78 (44.1)	28 (38.4)	0.89
**Never smoker**	99 (55.9)	45 (61.6)	
**Body mass index**, mean (SD), kg/m^2^	25.7 (4.2)	26.0 (3.4)	0.39
**Plasma lipids**, median (5th-95th percentiles) or mean (SD), mmol/L			
**Triglycerides**	0.95 (0.45–2.31)	1.04 (0.51–1.95)	0.91
**HDL-Cholesterol**	1.77 (0.52)	1.82 (0.59)	0.93
**LDL-Cholesterol**	3.82 (1.02)	3.77 (0.89)	0.58

NAT2 Study, n = 250, 2003–2005.

CNV: choroidal neovessels; HDL: high density lipoprotein; LDL: low density lipoprotein; SD: standard deviation.

^a^p for Student’s t test for age, Pearson Chi^2^ test for gender and logistic regression adjusted for age, gender and treatment for other variables.

**Table 3:** Associations between genetic polymorphisms and occurrence of CNV.

	Occurrence of CNV		Model 1^a^			Model 2^b^		
Genetics polymorphisms	No n = 177	Yes n = 73						
	n	n	HR	p-value	Global p-value^c^	HR	p-value	Global p-value^c^
	(%)	(%)	(95% CI)			(95% CI)		
***CFH* Y402H**					*0*.*40*			*0*.*37*
**TT (non-risk)**	41	17	Reference			Reference		
	(23.2)	(23.3)						
**CT**	83	32	0.97	*0*.*92*		1.06	*0*.*86*	
	(46.9)	(43.8)	(0.54–1.76)			(0.57–1.94)		
**CC**	53	24	1.29	*0*.*43*		1.33	*0*.*39*	
	(29.9)	(32.9)	(0.69–2.40)			(0.69–2.54)		
***ARMS2* A69S**					*0*.*07*			*0*.*09*
**GG (non-risk)**	52	14	Reference			Reference		
	(29.3)	(19.2)						
**GT**	82	38	1.68	*0*.*10*		1.61	*0*.*14*	
	(46.3)	(52.0)	(0.91–3.12)			(0.86–3.00)		
**TT**	43	21	1.78	*0*.*10*		1.83	*0*.*09*	
	(24.3)	(28.8)	(0.90–3.52)			(0.91–3.68)		

CI: confidence interval; CNV: choroidal neovessels; HR: Hazard ratio.

^a^Cox model adjusted for age, gender and treatment. Each variant was introduced in separate model.

^b^Model 2: Cox model adjusted for age, gender, treatment, BMI, smoking, HDL, triglycerides, genetic polymorphisms of *ARMS2* A69S or *CFH* Y402H.

^c^Global p-value was obtained using the genotype of the variant as a continuous variable according to the number of risk allele.

**Table 4:** Effect of DHA treatment on occurrence of CNV according to *CFH* Y402H (rs1061170) and *ARMS2* A69S (rs10490924) genotypes.

	Placebo group		DHA group			Interaction SNP*treatment		
	% of CNV	HR	% of CNV	HR	p-value	HR	p-value	Threshold for Bonferroni correction
		(95% CI)		(95% CI)		(95% CI)		
***CFH* Y402H**								
**Model 1** ^**a**^						0.43	*0*.*01*	*<0*.*025*
						(0.23–0.83)		
**TT (non-risk)**	38.2	1.0	16.7	0.25	*0*.*02*			*<0*.*017*
		-		(0.08–0.79)				
**CT**	26.0	1.0	29.2	1.11	*0*.*79*			*<0*.*017*
		-		(0.53–2.30)				
**CC**	23.1	1.0	39.5	2.09	*0*.*09*			*<0*.*017*
		-		(0.90–4.89)				
**Model 2a** ^**b**^						0.45	*0*.*02*	*<0*.*025*
						(0.24–0.87)		
**TT (non-risk)**		1.0		0.14	*0*.*008*			*<0*.*017*
		-		(0.03–0.59)				
**CT**		1.0		1.19	*0*.*65*			*<0*.*017*
		-		(0.56–2.51)				
**CC**		1.0		2.33	*0*.*06*			*<0*.*017*
				(0.98–5.55)				
***ARMS2* A69S**								
**Model 1** ^**a**^						0.69	*0*.*26*	*<0*.*025*
						(0.37–1.32)		
**GG (non-risk)**	15.6	1.0	26.5	1.88	*0*.*27*			*<0*.*017*
		-		(0.62–5.71)				
**GT**	30.0	1.0	33.3	0.91	*0*.*79*			*<0*.*017*
		-		(0.47–1.78)				
**TT**	38.7	1.0	27.3	0.74	*0*.*50*			*<0*.*017*
		-		(0.30–1.78)				
**Model 2b** ^**c**^						0.69	*0*.*26*	*<0*.*025*
						(0.36–1.33)		
**GG (non-risk)**		1.0		1.93	*0*.*27*			*<0*.*017*
		-		(0.61–6.16)				
**GT**		1.0		0.79	*0*.*49*			*<0*.*017*
		-		(0.40–1.56)				
**TT**		1.0		0.60	*0*.*32*			*<0*.*017*
		-		(0.22–1.62)				

CNV: choroidal neovessels; DHA: docosahexaenoic acid; CI: confidence interval; HR: hazard ratio; SNP: single nucleotide protein.

^a^Model 1: Cox model adjusted for age and gender, n = 250.

^b^Model 2a: Cox model adjusted for age, gender, BMI, smoking status, HDL- cholesterol, triglycerides and genetic polymorphisms of *ARMS2* A69S, n = 250.

^c^Model 2b: Cox model adjusted for age, gender, BMI, smoking status, HDL-cholesterol, triglycerides and genetic polymorphisms of *CFH* Y402H, n = 250.

**Table 5:** Variations of EPA+DHA in serum and red blood cells membranes according to *CFH* Y402H and *ARMS2* A69S at baseline and after 3 years DHA supplementation.

		EPA+DHA at baseline			EPA+DHA after 3 years DHA supplementation	
		(% fatty acids)			(% fatty acids)	
Genetic polymorphisms	n	Serum	RBCM	n	Serum	RBCM
		Median (5^th^-95^th^ percentiles)	Median (5^th^-95^th^ percentiles)		Median (5^th^-95^th^ percentiles)	Median (5^th^-95^th^ percentiles)
***CFH* Y402H**						
**TT**	58	2.0 (0.9–3.4)	3.9 (1.9–5.8)	57	2.4 (1.1–5.1)	4.8 (2.7–8.8)
**CT**	115	1.9 (1.0–3.9)	3.8 (2.2–5.9)	107	3.1 (1.3–5.8)	5.4 (3.0–8.3)
**CC**	77	1.9 (1.1–4.0)	3.6 (2.1–6.1)	69	3.1 (1.2–6.6)	5.4 (3.1–9.2)
**P anova**		*0*.*84*	*0*.*47*		*0*.*19*	*0*.*55*
***ARMS2* A69S**						
**GG**	66	1.9 (1.0–4.0)	3.7 (2.1–5.9)	64	3.0 (1.1–5.4)	5.2 (2.9–8.2)
**GT**	120	2.0 (1.1–3.7)	3.8 (2.0–5.7)	110	3.2 (1.2–6.0)	5.2 (2.9–8.5)
**TT**	64	1.9 (1.0–3.1)	3.8 (2.5–6.0)	59	2.7 (1.0–5.9)	5.3 (2.9–9.2)
**P anova**		*0*.*50*	*0*.*86*		*0*.*72*	*0*.*94*

NAT2 Study, n = 250, 2003–2005.

RBCM: red blood cells membranes.

## Results

**Fig 1:**
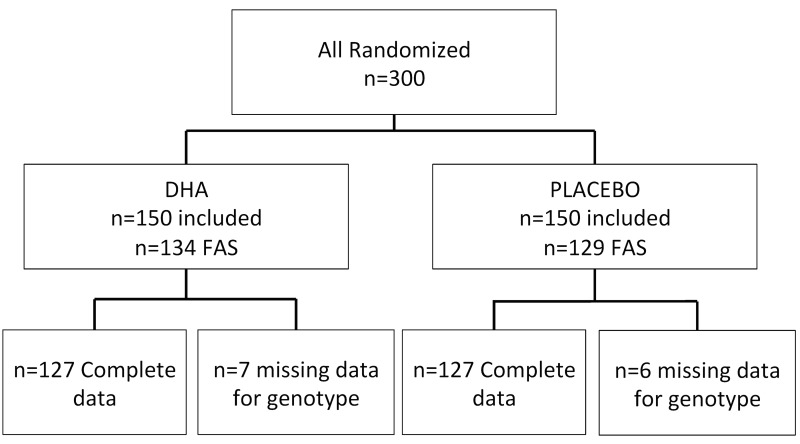
Diagram showing the population of Nutritional AMD Treatment 2 Study design included in this paper. Full analysis set (FAS) included patients having at least one post baseline value regarding occurrence of CNV. AMD: age-related macular degeneration; CNV: choroidal neovascularization; DHA: docosahexaenoic acid.

Among the 300 patients enrolled into the NAT2 study, 263 had at least one follow up assessment of CNV and could be included in the full analyses set (FAS). Among these 263 patients, seven had no data for any genotype and six had missing data for occurrence of CNV, mostly due to geographic atrophic development, which prevents evaluation of CNV occurrence. Thus, 250 patients were included in the analysis: 127 received DHA and 123 received placebo (Fig 1).

Table 1 reports baseline demographic, lifestyle and genetic risk factors for AMD according to treatment group (DHA or placebo). In our study, there was no statistically significant difference between treatment groups for gender (p = 0.08), age (p = 0.28), smoking status (p = 0.19), BMI (p = 0.47), plasma triglycerides (p = 0.23), HDL-cholesterol (p = 0.31) and LDL-cholesterol (p = 0.42). The distribution of *CFH* Y402H and *ARMS2* A69S polymorphisms was not statistically significant different between treatment groups (p = 0.16, and p = 0.97, respectively). Occurrence of CNV was the same between the two treatment groups: 29.9% in DHA group versus 28.5% in placebo group (p = 0.80).

Among the 250 patients with unilateral neovascular AMD, 73 (29.2%) developed CNV in the study eye. As shown in Table 2, patients who developed CNV were not different regarding age (p = 0.47), gender (p = 0.21), smoking status (p = 0.89), BMI (p = 0.39) and plasma lipids, compared to patients who did not develop CNV.

Table 3 displays associations between *CFH* Y402H and *ARMS2* A69S polymorphisms and occurrence of CNV adjusted for age, gender and treatment in model 1; plus BMI, smoking status, HDL-cholesterol, triglycerides and *ARMS2* A69S or *CFH* Y402H polymorphisms in model 2. Patients with the risk allele (C) for *CFH* Y402H had no statistically significant increased risk to develop CNV in the study eye (HR = 0.97 95%CI: 0.54–1.76 for heterozygotes and HR = 1.29; 95%CI: 0.69–2.40 for homozygotes). For *ARMS2* A69S polymorphism, patients with the risk allele (T) had no statistically significant risk to develop CNV in the study eye (HR = 1.68; 95%CI: 0.91–3.12 for heterozygotes and HR = 1.78; 95%CI: 0.90–3.52 for homozygotes).

Table 4 shows the effect of DHA treatment on occurrence of CNV according to *CFH* Y402H and *ARMS2* A69S polymorphisms, adjusted for age and gender in model 1, plus BMI, smoking, HDL-cholesterol, triglycerides and genetic *ARMS2* A69S or *CFH* Y402H polymorphisms in model 2. A significant interaction was observed between *CFH* Y402H and DHA supplementation (p = 0.01 in model 1 and p = 0.02 in model 2). No significant interaction was found between DHA supplementation and *ARMS2* A69S (p = 0.26 in model 1 and p = 0.27 in model 2). Among patients homozygous for the non-risk allele (T) for *CFH* Y402H, 38.2% in the placebo group developed CNV, compared to 16.7% in the DHA group. Of those patients homozygous for the risk allele (C), 23.1% in the placebo group developed CNV, compared to 39.5% in the DHA group. Among patients with CT genotype, 26.0% in the placebo group developed CNV, compared to 29.2% in the DHA group. There was a significant protective effect of DHA among patients homozygous for the *CFH* Y402H non-risk allele (Model 2: HR = 0.14; 95%CI: 0.03–0.59; p = 0.008) but no significant effect of DHA among those homozygous (Model 2: HR = 2.33; 95%CI: 0.98–5.55; p = 0.06) or heterozygous for the risk allele (Model 2: HR = 1.19; 95%CI: 0.56–2.51; p = 0.65).

Table 5 reports variations of EPA+DHA in serum and in RBCM according to genotype. We found no difference in serum or RBCM EPA+DHA among *CFH* Y402H and ARMS2 A69S polymorphisms at baseline as well as after 3-years DHA-supplementation.

## Discussion

This study’s major finding was evidence of a possible interaction between *CFH* Y402H polymorphism and the preventive effect of DHA supplementation. This study showed a protective effect of DHA supplementation only among patients homozygous for the *CFH* Y402H non-risk allele (T). For the non-risk allele group, occurrence of CNV was 38.2% in placebo group versus 16.7% in DHA group (p = 0.008). No statistically significant effect of DHA supplementation was observed in patients with at least one risk allele for *CFH* Y402H polymorphism (CT and CC). No significant interaction was found between DHA supplementation and *ARMS2* A69S genotype. These results suggest that the association between DHA supplementation and occurrence of CNV might be moderated by genetic susceptibility, particularly related to the *CFH* Y402H variant. These results imply that the genetic predisposition to AMD conferred by *CFH* Y402H may limit the benefits provided by DHA supplementation.

To our knowledge, no other study has explored interactions between DHA supplementation and genetic polymorphisms for the occurrence of CNV. AREDS has explored interactions of supplementation with antioxidants and zinc with genetic polymorphisms on progression to advanced AMD, but results are not consistent. In 2008, one study [31] showed that among subjects with antioxidant plus zinc supplementation (versus placebo) and among subjects with zinc supplementation (versus no zinc supplementation), subjects with a non-risk allele for the *CFH* gene had a reduced risk of progression to advanced AMD compared to subjects carrying at least one risk allele. This study did not find any interaction between supplementation and *ARMS2* A69S polymorphism. Recently, another AREDS study [38], found that *CFH* and *ARMS2* A69S polymorphisms did not significantly modify the benefit of the AREDS supplements. On the other hand, some studies have explored interactions of dietary intake of omega-3 fatty acids with genetic polymorphisms for risk of AMD [23, 29, 32]. Reynolds et al. [23] found a protective effect of dietary intake of DHA for geographic atrophy among subjects with homozygous non-risk allele for the *CFH* gene (p = 0.02). No significant association was found for the homozygous risk allele (p = 0.37) but the interaction was not significant (p = 0.16). For *ARMS2* A69S, authors found a protective effect of DHA intake for GA among subjects homozygous for the risk allele but the interaction was not significant (p = 0.05). Our results are consistent with this study; although our results concern the alternate form of late AMD, the occurrence of CNV.

In the Alienor study, a significant association between *CFH* Y402H and plasma long chain omega-3 PUFAs was reported. Subjects carrying the homozygous risk allele for *CFH* Y402H had lower plasma long chain omega-3 PUFAs levels (p = 0.005) [27], but the interaction between *CFH* Y402H and plasma omega-3 PUFAs with regard to AMD risk was not statistically significant [27]. In the NAT2 study, we had serum and RBCM EPA+DHA data at baseline and after 3-year DHA-supplementation, but we did not find any difference in serum or RBCM EPA+DHA according to *CFH* Y402H and *ARMS2* A69S polymorphisms at baseline as well as after 3 years DHA-supplementation.

CFH is a key regulator of complement by inhibiting the alternative pathway. The risk allele appears to impair this regulatory function of CFH, leading to complement over activation, thereby increasing the risk of AMD [39, 40]. EPA and DHA have anti-inflammatory, antiapoptotic, and antiangiogenic effects [41], and previous studies have shown that higher intake of EPA+DHA were associated with reduced serum levels of C-reactive protein [42, 43]. It is plausible that EPA+DHA reduced the risk of AMD by modulating the immune response and inflammatory response [41] with a stronger effect among subjects with non-risk allele for *CFH* gene.

In the present study, age and gender were not significantly associated with occurrence of CNV, nor were any of the environmental factors usually associated with AMD [2] (smoking status, BMI, plasma lipids). These results suggest that environmental factors could play a minor role in bilateralism of exudative AMD. Surprisingly, in our sample *CFH* Y402H and *ARMS2* A69S polymorphisms were not significantly associated with the occurrence of CNV in the study eye (p = 0.16, and p = 0.97, respectively), which might be explained by the fact that AMD is a bilateral disease and all patients were already affected by exudative AMD in one eye. These risk factors are commonly associated with onset and progression of AMD and not with the onset of AMD in the second eye. The allelic frequencies of *CFH* Y402H and *ARMS2* A69S, calculated from data in Table 1, were 0.538 and 0.496, respectively. These results are similar to those previously published in Caucasian subjects with exudative AMD [9, 35, 44–46].

Despite the lack of global effect of DHA on occurrence of CNV, our results suggest that the benefit of DHA supplementation could be optimized in a genetically defined population. This study contributes to personalized approach for DHA supplementation in prevention of exudative AMD.

The strengths of this study include its double-blind, prospective and randomized design, and the well-defined and homogenous group of neovascular AMD patients. A potential limitation could be the relatively small number of patients who developed CNV (n = 73), which could affect detection of an interaction between genes and DHA supplementation, particularly for *ARMS2* A69S polymorphism. Although the interaction term between the *CFH* variant and treatment was significant, a potential lack of power, reflected by the wide confidence intervals, cannot be overlooked. These results should be considered with caution, however, due to unplanned and retrospective analyses.

In conclusion, we showed a suggestive protective effect of DHA supplementation among patients with the *CFH* Y402H homozygous non-risk allele. These results may imply that a genetic predisposition to AMD conferred by the *CFH* Y402H polymorphism limits the benefits provided by DHA supplementation, and might explain the limited benefit of DHA supplementation for occurrence of CNV in NAT2 or AREDS2 studies. Further fundamental studies are necessary to understand the exact mechanisms by which genetic and environmental factors interact to promote the development and the progression of AMD.

## Supporting Information

S1 File NAT2 IRB approval. (PDF)

S2 File NAT2 CONSORT check list. (PDF)

S3 File NAT2 data set used for analysis. Idnat2: id for each patient; tg1: triglycerides; Hdl-c1: high density lipoprotein at baseline; Ldl-c1: low density lipoprotein at baseline; epadhas1: eicosapentaenoic acid and docosahexaenoic acid in serum at baseline; epadharcmc1: epa and dha in red blood cell membranes at baseline; epadhas5: epa and dha in serum at the last visit; epadharbcm5: epa and dha in red blood cell membranes at the last visit; TREAT: DHA-supplementation 0 = placebo, 1 = DHA; cnv: presence of choroidal neovessels 0 = no, 1 = yes; CFH: complement factor H genotype; ARMS2: age-related macular susceptibility 2 genotype; TABAC: smoking use 0 = never smoker, 1 = ever smoker; BMI: body mass index.
(PDF)
