# Supplementary material for: CFH Y402H and ARMS2 A69S Polymorphisms and Oral Supplementation with Docosahexaenoic Acid in Neovascular Age-Related Macular Degeneration Patients: The NAT2 Study
Source: PLoS One. 2015 Jul 1;10(7):e0130816. doi: 10.1371/journal.pone.0130816 (PMC4489493; doi:10.1371/journal.pone.0130816)
Supplement: S1 File — (PDF) [file pone.0130816.s001.pdf]

COMITE CONSULTATIF POUR LA PROTECTION DES PERSONNES DANS LA RECHERCHE BIOMEDICALE
ILE DE FRANCE - PARIS - St ANTOINE

Président :
V.G. LEVY
Vice-Président :
C. GRICOURT
Secrétaire général :
J.L. PRUGNAUD
Secrét. général adjoint :
F. BERGIER DESCOMBES
Trésorier :
G. ETIENNE
Trésorier adjoint :
B. NALPAS

Monsieur le Docteur E. SOUIED
Clinique ophtalmologique
Universitaire de Créteil
40, avenue de Verdun
94010 CRETEIL Cedex

Projet de recherche biomédicale enregistré sous le numéro: 03562

Le Comité a été saisi le 13 juin 2003

Par Monsieur le Docteur Eric SOUIED

du projet de recherche biomédicale suivant: **(Recherche Avec Bénéfice Individuel Direct).**

Etude comparative, parallèle, randomisée, en double insu d'un supplément nutritionnel oral contenant du DHA (acide docosahexaenoique) versus placebo dans la prévention de la dégénérescence maculaire liée à l'âge. Etude NAT 2.

Dont le promoteur est : **Bausch&Lomb/Chauvin**

Le Comité a examiné les informations relatives à ce projet lors de la séance du mardi 1er juillet 2003.

Les rapporteurs mandatés étaient: Mme SCHIMPFF, Mme ROSENZWEIG

(*) Ont participé à la délibération : Mmes DEBRU(5), DELZANT(1), DUBOULOY(4), KURTZ(7), SCHIMPFF(1).
MM. ETIENNE(3), LEVY(1), NALPAS(1), POUILLARD(2),PRUGNAUD(3).
(1) Médecins, recherche bio-médicale, (2) Médecins, (3) Pharmaciens,
(4) Infirmières, (5) Ethique, (6) Social, (7) Psychologue, (8) Juridique

Le Comité a adopté la décision suivante : Avis favorable, les modifications demandées ayant été reçues.

FAIT A PARIS, le 22 juillet 2003

Le Président

ace europe

ACE Insurance S.A.-N.V.
Le Colisée
8, avenue de l'Arche
92419 Courbevoie Cedex

33 (0)1 55 91 45 45 *tél*
33 (0)1 47 88 45 10 *fax*
www.aceeurope.com

ATTESTATION D'ASSURANCE

RESPONSABILITE CIVILE

PROMOTEUR DE RECHERCHES BIOMEDICALES

Nous soussignés, **ACE Insurance S.A – N.V**, Immeuble le Colisée 8, avenue de l'Arche, 92419 Courbevoie Cedex attestons que :

LABORATOIRES CHAUVIN
Parc Millénaire II
416, rue Samuel Morse
B.P 1174
34009 MONTPELLIER CEDEX

agissant en tant que PROMOTEUR,

est assuré par un contrat de Responsabilité Civile référencé **5.323.978** souscrit à effet du **1er Juin 2003** et conforme aux dispositions légales et réglementaires françaises sur les recherches bio-médicales et notamment aux dispositions de la loi n° 88.1138 du 20.12.88, modifiée par la loi n° 90-86 du 23.01.90, du décret n° 91.440 du 14.05.91 et par la loi n° 94.630 du 25.07.1994.

Titre de la recherche assurée :

Double-masked, randomised, parallel, comparative study of oral supplementation with DHA (Docosahexaenoic acid) versus placebo in the prevention of age-related macular degeneration. NAT 2

**Référence de l'étude**	**: NAT 2**
**Phase**	**: NA**
**Nombre de patients**	**: 350**
**Date de début de l'étude**	**: octobre 2003**
**Date prévisionnelle de fin de l'étude**	**: Décembre 2008**

LIMITES DE GARANTIE :

762.245 Euros par Victime.
4.573.471 Euros par protocole de recherche.
7.622.451 Euros par année d'assurance.

ace europe

ACE Insurance S.A.-N.V.
Le Colisée
8, avenue de l'Arche
92419 Courbevoie Cedex

33 (0)1 55 91 45 45 *tél*
33 (0)1 47 88 45 10 *fax*
www.aceeurope.com

La garantie est conforme à l'obligation d'assurance instituée par les textes de loi précités, article L 209-7 à la charge du promoteur, tant pour sa propre responsabilité que pour celle des intervenants.

La présente attestation est délivrée pour être remise au Comité Consultatif de Protection des Personnes.

Fait à Courbevoie, le 11 avril 2003

Siège Social : 9-31 Avenue Nerviens - 1040 Bruxelles - Belgique - S.A. au capital de € 146.721.272,54
Autorité de contrôle : Office de Contrôle des Assurances - 61 avenue de Cortenbergh - 1000 Bruxelles - Belgiqu
Direction Générale pour la France : 8, avenue de l'Arche - 92419 Courbevoie Cedex
RC. 325 743 904 Nanterre B - N° d'identification 325 743 904 00139 - APE 660 E
