# Supplementary material for: CFH Y402H and ARMS2 A69S Polymorphisms and Oral Supplementation with Docosahexaenoic Acid in Neovascular Age-Related Macular Degeneration Patients: The NAT2 Study
Source: PLoS One. 2015 Jul 1;10(7):e0130816. doi: 10.1371/journal.pone.0130816 (PMC4489493; doi:10.1371/journal.pone.0130816)
Supplement: S3 File — Idnat2: id for each patient; tg1: triglycerides; Hdl-c1: high density lipoprotein at baseline; Ldl-c1: low density lipoprotein at baseline; epadhas1: eicosapentaenoic acid and docosahexaenoic acid in serum at baseline; epadharcmc1: epa and dha in red blood cell membranes at baseline; epadhas5: epa and dha in serum at the last visit; epadharbcm5: epa and dha in red blood cell membranes at the last visit; TREAT: DHA-supplementation 0 = placebo, 1 = DHA; cnv: presence of choroidal neovessels 0 = no, 1 = yes; CFH: complement factor H genotype; ARMS2: age-related macular susceptibility 2 genotype; TABAC: smoking use 0 = never smoker, 1 = ever smoker; BMI: body mass index. (PDF) [file pone.0130816.s003.pdf]

idnat2	tg1	hdl_c1	ldl_c1	epadhas1	epadharbc	epadhas5	epadharbc	TREAT
1	1.75	2.6	4.95	1.9	4.8	6.425124	9.593986	1
2	0.92	3.67	3.06	1.8	3.6	4.740707	8.032918	1
3	2.07	1.28	5.95	1.7	4.4	0.900128	3.570385	0
4	1.37	1.51	3.47	2.1	4.7	2.72924	5.07172	0
5	0.85	1.22	4.35	2.2	4	1.836421	4.287468	0
6	1.32	1.59	4.93	1.7	4.9	3.730833	4.54886	1
7	1.57	1.31	4.96	1.9	4	1.15735	4.495858	0
8	1.45	1.27	4.94	1.6	3.8	3.000864	6.562664	1
9	1.57	0.95	6.06	1.4	3.5	0.716598	3.109495	0
10	1.43	2.03	3.73	2.2	3.7	3.1315	5.393767	1
12	0.62	2.07	3.33	1.8	4.3	1.721036	3.440048	0
13	0.71	1.94	4.41	1.5	3.4	3.863	6.526	1
14	1.34	1.04	3.3	1.9	4.7	2.847	5.183	0
15	0.79	1.56	7.9	1.8	2.9	3.571207	7.634886	1
16	0.75	0.89	3.69	1.3	4	1.365428	3.664564	0
17	1.16	1.46	3.25	1.7	4.2	1.394177	4.687056	0
18	1	1.62	3.63	2.5	4.5	1.152148	3.275277	0
19	1.95	1	4.82	1.3	5.6	4.259715	8.136066	1
21	0.83	1.67	3.45	1.9	3	2.034215	4.968078	1
22	0.88	0.99	4.78	2.1	4.4	1.670896	4.959265	0
23	1.29	2.15	5.71	1.9	5.3	2.118318	7.016832	1
24	0.48	2.34	3.78	2.9	5.3	1.595373	3.545398	0
25	0.75	1.15	2.48	5.8	5.5	2.404675	4.996608	0
26	2.05	1.17	3.54	2.3	4.1	2.053232	5.210252	1
27	1.19	1.27	3.66	1.6	5.5	1.233416	3.764514	0
28	0.7	2.4	2.59	1.4	3.1	1.192187	2.651749	1
29	1.39	2.04	3.39	1.9	5.4	1.587562	2.504725	0
30	1.8	1.88	7.05	1.5	4.2	2.687932	4.610372	1
31	0.69	1.53	4.14	2.3	4.4	1.842538	3.305597	0
32	0.45	2.37	3.13	2.5	5.9	3.153532	4.208435	1
33	0.62	1.92	3.94	5.2	6.8	4.350154	4.339	0
34	1.41	2.73	5.86	1.9	4.9	1.56381	3.765099	0
35	0.76	1.48	3.77	2.1	4.2	4.143931	7.358682	1
36	0.5	1.02	3.57	3.5	6.4	5.125645	8.703557	1
37	1.45	1.15	5.3	2.9	6.1			1
38	1.23	0.86	4.21	1.7	4.8	3.682398	6.125122	0
39	1.05	1.4	4.78	2.6	5			1
41	1.54	0.71	2.87	2.4	4.3	2.38239	6.326948	1
42	1.1	1.52	4.75	1.9	3.5	1.541995	2.853099	0
43	0.56	2.66	2.97	3.6	5.2	2.20475	4.307804	0
44	1.06	2.04	4.04	1.6	4.3	4.613674	7.600223	1
48	0.65	1.11	4.77	3.1	5	2.296883	5.594227	0
50	1.38	1.17	2.29	2.1	5.7	3.387121	8.190334	1
51	1.46	1.3	3.75	3.7	5.8	7.3257	5.998034	0
52	1.24	1.32	5.29	2	5.2	5.540209	9.209639	1
53	0.72	1.63	3.47	2.3	4.3	1.657914	4.880632	0
54	1.03	1.97	4.66	1.7	4.6	3.260518	7.223338	1
55	0.82	1.28	3.97	1.2	3.8	1.373862	2.90724	0
56	1.52	0.96	4.64	1.6	2.8	1.612849	4.73519	1
57	0.95	1.66	3.89	1.7	3.6	1.71064	3.622102	0
58	0.9	1.07	5.08	2.2	5.6	2.386166	6.138648	0

59	1.4	1.34	4.74	3.2	5.1			1
60	0.49	2.64	3.04	2.8	4.9	4.506487	7.434927	1
61	1.07	2.12	3.07	3.1	5.9	3.223834	4.614152	0
62	0.58	1.99	4.23	2	3.2	2.467045	5.878537	0
63	0.8	1.16	4.29	2	4.1	2.74831	5.386194	1
64	1	1.58	5.37	2.2	3.9	4.678499	8.777464	1
66	0.5	1.74	3.64	1.4	2.2	4.500437	7.082959	1
67	0.46	1.99	3.59	2.7	3.4	2.022617	4.356431	0
68	1.15	1.21	5.35	2.3	3.5	5.282904	9.194794	1
69	0.62	2.27	3.35	3.4	4			0
70	1.01	1.37	6.37	3.4	3.2	4.158435	7.6343	1
71	0.54	1.73	3.95	2.8	1.9	3.492312	8.51369	1
72	1.79	1.36	6	2.4	5	1.459783	3.382987	0
74	1.27	0.85	3.99	2.4	4.9	2.973321	5.094925	0
76	0.97	1.98	5.15	1.9	2.1	4.8	9.9	1
77	0.67	1.09	4.06	1.7	3.7	1.373045	2.666149	0
78	0.7	1.37	3.99	2.9	6.6	3.227044	6.930944	1
79	1.94	1.78	4.66	1.4	4.6	0.967025	5.128205	0
81	2.95	1.5	4.13	1.9	5.4	3.31773	7.903377	1
82	0.38	2.56	3.23	3.4	6.2	6.198274	8.833962	1
84	0.4	2.16	3.27	3.7	5.2	3.732054	5.331529	0
87	0.74	1.08	4.51	2.2	5.2	4.252387	6.897138	1
88	0.69	1.74	3.11	2.4	4.8	2.127701	3.828281	0
89	1.01	0.99	4.47	2.5	5.7	2.155888	4.929596	0
90	0.82	1.57	5.94	3.1	5.4	1.487	3.910723	0
91	0.49	2.3	4.34	4	6.2	7.014133	7.899926	1
92	1.25	1.46	2.35	1.7	4.9	3.837765	6.174066	1
93	1.32	1.08	3.77	2.7	6	2.294111	4.810651	0
95	1.14	0.98	4.73	1.3	4.4			0
96	1.13	1.86	3.69	2.6	3.5	4.45473	7.575283	1
97	1.13	1.84	2.78	3.1	7.5	3.237	5.687	1
99	1.12	2.32	4.03	1.8	4.5	1.552941	2.981019	0
101	0.6	2.02	3.54	2.2	3.7	2.409556	3.753425	0
102	1.28	1.33	3.56	2.1	3.5	1.30668	3.334602	0
104	2.17	1.41	4.64	1.9	4.3	5.878537	9.030233	1
105	1.19	1.77	3.52	1.5	3.6	3.587558	5.264367	1
106	2.12	1.93	3.54	3	6	2.586463	4.25236	1
107	0.41	3.15	2.83	2.7	4.9	1.165325	2.978088	0
108	1.19	1.42	3.3	2.3	3.3	3.726838	5.042856	0
109	0.81	2.27	3.87	2.6	3.7	7.031872	9.402916	1
111	1.01	1.7	4.45	1.5	2.8	1.045943	4.667938	0
112	1.4	1.79	3.51	1.4	3.2	0.981783	2.471405	0
113	1.11	2.39	3.76	2.3	3.6	1.94958	3.717195	0
114	1.17	2.26	3.18	2.5	4.7	4.605931	6.302436	1
115	1.47	1.67	4.81	1.9	5.3	3.455847	5.595555	1
116	0.61	2.77	2.69	2.6	4.5	2.553488	4.921034	0
117	0.76	2.95	4.81	2.4	4.1	3.966705	7.659328	1
119	0.55	1.75	2.73	1.6	4.3	5.163102	7.719116	1
120	1.43	1.74	3.74	1.6	4.9	3.122215	5.637169	0
121	3.33	1.35	3.61	4.2	2.7	4.092224	6.095578	0
122	1.3	2.11	3.15	1.2	2.6			1
124	0.7	2.07	3.39	2.5	4.8	9.849065	6.561817	0

125	1.04	2.54	4.08	3.1	5.5	4.837528	8.161678	1
126	0.83	1.75	3.54	2.2	2.5	2.2735	5.512665	0
127	0.58	2.59	2.9	1.6	3.3	4.741489	6.837016	1
128	0.44	2.38	3.75	1.8	2.9	3.020791	4.119834	0
129	0.87	2.84	4.32	2	3	2.264605	3.619065	0
130	1.16	3.04	3.91	2.4	3.7	3.930536	7.445358	1
131	0.69	2.35	2.63	2.6	2.6	4.117385	4.252823	0
135	1	2.05	4.6	1.3	2.3	4.221531	6.585167	1
136	0.97	1.84	3.78	2.3	4.2	3.267241	6.786363	0
137	0.68	1.73	2.24	1.3	3	1.300085	3.143399	0
138	2.04	1.92	4.03	0.9	1.8	1.331869	3.389915	0
139	0.66	1.99	2.77	1.4	2	4.180597	7.594673	1
140	1.2	1.52	4.17	1.2	2.4	3.055766	6.310398	1
141	1.75	1.39	4.06	1.2	1.6	3.632777	9.057903	1
142	0.52	2.4	3.18	2.3	3.1	6.056926	9.849065	1
144	0.38	2.52	2.24	7.5	5.7	2.72658	5.892215	0
147	2.17	1.32	3.16	1.6	3.2			0
148	1.16	1.96	4.03	2.5	4.1	3.716355	6.496948	1
149	0.61	2.23	4.01	4.4	4.5	4.219377	7.348724	1
150	0.82	2.83	3.96	1.7	3	2.076	4.384	1
151	0.78	1.29	5.02	2	3.1			0
152	0.92	1.88	2.96	1.4	2.8	1.553602	2.690087	0
154	0.5	2.42	2.84	1.3	3.2	2.45839	4.574859	1
155	0.95	2.12	4.68	1.3	3.9	1.132473	3.269861	0
157	1.01	2.66	2.74	3.5	5.3	3.93864	3.755525	1
158	0.67	2.87	3.61	2	4.1	1.788482	5.34518	0
159	2.31	1.51	4.77	1.1	2.4	4.665952	4.187134	1
161	0.56	1.66	4.54	2.1	3.1	3.909243	5.771335	1
162	0.68	2.2	5.7	1.7	2.3	4.884171	8.294449	1
164	0.74	1.23	2.64	1.3	3.5	1.600105	3.354991	0
165	2.97	1.36	5.05	1.2	1.8			0
166	0.57	2.11	3.19	1.4	3.4	1	5.6	1
167	0.75	3.3	3.99	2.5	4	2.070175	4.49255	0
168	0.84	2.75	3.74	1.6	4.6	3.589118	6.534607	1
169	1.64	2.16	4.21	2	1.8			0
170	0.47	1.63	3.34	1.5	3.4	3.3	4.039102	1
171	0.77	1.53	4.37	2.4	4.5	4.1	6.384838	1
172	0.51	1.9	3.87	1.2	3.2	1.990807	2.547834	0
173	1.12	2.21	3.86	1.3	2.9	3	4.255822	1
174	0.82	1.53	4.23	1	2	1.1	3.027996	0
175	0.8	2.31	3.03	0.9	1.9	1.886706	3.945312	0
176	0.64	2.1	3.02	2.4	3.6	3.058596	5.733482	1
177	0.77	2.48	3.16	1.1	2.2	1.548662	2.309972	0
178	0.89	2.05	5.59	2.3	3.7	2.229498	2.493033	0
179	2.46	1.19	6.01	0.9	1.5	3.258743	3.877809	1
180	1.2	1.27	2.7	1.3	3.2	4.422603	3.084115	1
181	0.73	1.42	3.04	2.9	4.6	2.760786	5.195857	0
182	0.41	2.48	3.7	1.8	3.4	4.211284	5.645922	1
183	1.86	2.23	2.97	1.5	2.9	2.243692	4.735959	0
186	1.74	1.52	3.26	1.7	3.8	1.558944	5.069195	0
187	1.08	1.8	3.28	1.9	3.2	2.067985	4.107585	0
188	0.57	2.54	2.67	2.2	4.1			1

189	3.37	1.3	4.21	1	2.7	2.03603	4.721446	1
191	1.41	1.04	3.21	2	4.9	2.061463	5.760277	0
192	1.03	1.72	4.83	2.6	4.7	3.001654	5.643155	1
194	2.46	1.93	4.99	1.7	4.5	2.048851	5.427779	1
195	1.21	1.45	4.38	1.3	3.8	0.913669	1.921411	0
197	0.98	1.7	3.54	2.7	5.3			1
198	1	1.42	2.43	1.6	3.4	2.147782	6.218123	1
199	1.39	1.34	4.71	4.1	6.2	2.792574	6.921837	0
202	0.75	1.55	3.62	3	5	1.813125	4.928089	0
203	1.76	1.47	4.51	2.8	4.7	4.517489	7.548802	1
204	0.74	2.75	3.25	2.5	3.5	1.207386	2.970074	0
205	1.03	1.67	5.11	2.2	5.4	5.996123	8.191805	1
206	0.87	1.7	3.26	1.9	3.8	3.299185	5.08243	0
208	0.81	2.94	3.83	2.6	4.8	6.668353	8.50535	1
209	1.11	1.84	3.45	1.4	3.7	6.102402	9.593817	1
211	0.77	3	3.44	2.8	4.1	2.239434	5.024737	0
212	1.05	2.02	3.42	2.8	4	4.092375	6.850353	0
213	0.93	2.07	3.38	2.8	4.8	4.264644	8.168009	1
214	1.43	1.48	5.63	1.9	3.6	3.079958	5.103631	0
215	0.37	2.04	2.13	1.3	2.7			0
216	0.93	2.16	2.53	2	3.2	6.619078	8.479341	1
217	0.75	1.65	5.51	2	3.8	5.055323	7.430832	1
218	1.43	1.07	3.92	2.8	3.7	1.877597	3.754459	0
219	1.47	2.12	3.73	1.8	4.2	1.807801	3.962786	0
220	2.48	1.33	3.12	1.4	3.5	3.725796	7.211279	1
221	1.12	2.1	3.73	2.2	3.6	2.011442	4.762053	0
222	1.17	1.4	2.83	2.7	4.4	2.037956	4.419339	0
223	2.03	1.5	5.74	4	4.4	4.28423	7.771658	1
224	0.93	1.23	3.57	0.9	1.8	3.795556	6.371164	1
225	1.63	0.93	2.77	1.9	4.1	3.019024	6.421571	0
226	0.9	1.75	2.86	2.7	5.2			1
228	0.96	1.53	2.91	1.1	2.6	5.355731	8.058122	1
230	0.79	1.1	2.16	1.9	3.5	4.557503	4.073538	1
231	1.22	1.54	4.13	2.2	3	3.759339	7.105125	1
232	0.74	1.82	3.45	1.6	2.7	1.999846	3.686332	0
233	1.32	1.48	3.07	1.7	3.5	2.007401	3.746928	0
234	0.45	2.2	2.44	4.8	5.1	2.207459	4.958004	0
235	0.97	1.33	4.5	1.4	3.1			1
236	1.75	1.46	3.02	1.2	2.3	3.23428	4.270439	1
237	0.45	1.92	2.22	2.4	3.2	3.63545	7.50805	1
238	1.69	1.14	4.5	1.9	4.1	2.332542	4.633711	1
240	1.46	1.85	3.09	1.3	3.5	2.603176	3.960212	0
241	1.15	1.12	3.2	2.4	4.4	4.358265	7.835499	1
242	1.27	1.92	5.06	1.4	4	4.683227	7.205324	1
243	0.71	1.79	2.58	1.1	3.5	2.186306	4.080445	0
244	2.83	0.61	2.43	0.7	3	0.968546	2.788925	0
245	0.68	2.31	2.76	1.8	1.5	4.680441	6.694954	1
246	2.45	0.89	4.22	1.1	2.8	1.911505	3.890964	0
247	0.8	3.07	2.64	0.8	2.3	3.858001	6.686338	1
248	1.1	1.62	2.59	0.9	3.1			0
249	0.88	2.11	3.88	1.6	4.3	5.583308	8.155124	1
251	0.77	2.09	3.04	2.2	5.4	5.787471	7.344405	1

252	0.87	2.08	3.16	2	5.1			0
253	0.79	1.72	3.61	1.7	3.8	1.877792	3.38186	0
254	0.5	1.55	2.94	2.1	3.4	3.258056	5.715088	1
255	0.51	1.53	2.41	1.9	3.6	3.131457	5.719103	1
256	1.68	1.45	4.66	1.5	3.2	1.478442	3.412556	0
258	0.64	1.58	1.96	2.4	4.5	1.186809	3.591393	0
259	0.94	2	3.19	1.7	3	3.313398	7.733983	1
260	0.75	1.35	3.25	1.6	3	3.219153	6.29029	1
261	1.52	1.79	3.35	2.1	3.4	2.408544	4.552407	0
262	0.87	1.92	2.3	2.1	2	2.054125	4.720343	1
263	0.51	2.08	4.23	1.6	3	1.576726	4.146319	0
265	0.67	1.63	3.88	1.1	3.3	1.532508	3.622754	0
266	1.45	1.28	4.96	1.3	2.9	2.309691	5.041803	1
267	1.07	2.35	3.59	1.5	2.8	2.156165	3.785621	0
268	0.61	2.31	4.43	2.2	3.5	4.349461	4.842225	1
269	1.15	1.78	4.31	1.1	2.5	2.397416	5.069003	0
270	0.4	2.05	2.45	2.2	2.9	4.566194	7.375637	1
272	2.91	1.09	3.03	2	3.9	3.313638	6.450326	1
273	0.76	2	1.72	0.9	5.6	4.784059	7.005378	1
275	1.41	1.69	4.9	1.8	5.1	2.209694	5.785479	0
276	1.86	1.28	3.21	1.4	3.7	3.536233	7.117577	1
277	0.86	1.66	4.22	1.8	3.8	3.134375	6.45729	1
278	1.82	1.11	3.35	0.9	2.6	1.32689	2.775811	0
279	0.6	2.51	4.32	1.2	2.7	2.850083	4.238237	0
280	0.7	1.68	3.78	1.8	2.7	3.354771	6.63583	1
281	1.87	1.59	3.86	1.4	2.8	3.956372	6.329966	1
282	0.81	1.06	3.7	2.2	5	3.941505	7.048404	1
283	1.01	1.61	3.26	0.7	2.5	1.541611	3.507581	0
284	1.12	1.94	3.8	3.7	5.8	3.992354	5.626809	0
285	1.54	1.66	2.89	1.7	3.3	1.750423	4.265649	0
286	0.59	1.85	2.1	2	3.9	4.565714	7.202367	1
288	0.55	2.32	3.64	2.8	5.2	4.097925	7.924357	1
289	0.95	2.4	4.93	1.7	3.5	3.396052	4.444742	0
290	0.92	1.55	5.09	2.4	4	3.946723	6.050887	1
292	0.96	1.91	5.02	1.6	3.6	3.737733	5.116666	0
293	0.99	1.69	2.87	3	4.9	2.951058	3.433535	1
294	1.56	1.92	3.77	1.4	2.5	2.8468	5.202558	0
295	1.04	2.55	3.3	1.3	2.2	3.95289	6.290258	1
296	0.87	2.12	2.77	3.9	4.4	2.179975	5.40164	0
297	1.18	2.34	2.31	1	2.1	2.160989	3.199134	0
298	3.51	0.85	3.97	1	2.6	1.693159	3.557003	0
299	1.63	1.65	4.2	1.2	3.1	3.092519	6.3025	1
300	0.93	2.73	1.74	1.5	3.6	5.386479	6.051012	1

sexe	age	Date_cnv	date_fin	CFH	ARMS2	TABAC	cnv	BMI
Female	83		12/14/2006	CC	GG	0	0	23.73324
Female	80	1/4/2006	1/4/2006	CT	TT	0	1	20.07733
Female	74		12/21/2006	CC	TT	0	0	23.53037
Male	71	6/6/2006	6/6/2006	CT	TT	1	1	24.7721
Male	82	1/20/2005	1/20/2005	CC	GT	0	1	20.43817
Female	79	11/3/2005	11/3/2005	TT	TT	0	1	24.65483
Female	65		1/18/2007	CC	GT	0	0	21.2585
Male	72		1/31/2007	CT	TT	1	0	23.38435
Female	80		1/31/2007	CT	GT	0	0	31.63458
Male	78		1/31/2007	CC	GG	1	0	24.21229
Female	78		2/28/2007	CC	GT	0	0	26.33289
Female	74		1/18/2007	CT	TT	1	0	32.28306
Male	81		1/23/2007	CT	GG	1	0	23.12467
Female	66		1/18/2007	CT	GT	0	0	29.29688
Male	72	9/21/2005	9/21/2005	CC	TT	1	1	20.76125
Female	77	10/10/2005	10/10/2005	TT	GT	0	1	27.4406
Female	74		2/21/2007	TT	TT	1	0	20.66116
Female	76	1/23/2007	1/23/2007	CC	TT	0	1	26.82742
Female	76		2/28/2007	CT	GT	0	0	23.11111
Female	84		2/21/2007	CT	GG	0	0	26.98962
Female	83		3/21/2007	CT	GG	0	0	
Female	77	1/10/2005	1/10/2005	TT	GT	1	1	24.69136
Male	79		2/28/2007	CT	GT	1	0	20.93664
Male	67		2/22/2007	TT	TT	0	0	25.39063
Male	67	11/15/2004	11/15/2004	CC	TT	1	1	25.39063
Female	72		3/28/2007	TT	GT	0	0	21.10727
Female	78		3/7/2007	TT	GT	0	0	19.11111
Male	63	10/14/2005	10/14/2005	CC	GG	1	1	21.71925
Female	69	12/8/2005	12/8/2005	TT	TT	0	1	20.93664
Male	76		3/13/2007	CT	GG	1	0	29.3724
Female	65	6/25/2004	6/25/2004	CC	GT	0	1	25.40282
Female	81		3/13/2007	CT	GT	0	0	22.09317
Female	73		3/8/2007	TT	GG	0	0	32.87197
Female	71		3/8/2007	TT	GT	0	0	23.66144
Female	72	9/27/2004	9/27/2004	CC	TT	0	1	48.2435
Male	68	1/15/2007	1/15/2007	TT	TT	1	1	30.48316
Male	68	6/21/2005	6/21/2005	CC	GT	1	1	23.14726
Male	83		3/15/2007	TT	GG	1	0	20.70082
Female	66		3/8/2007	TT	TT	1	0	28.66889
Female	66		3/21/2007	CC	GT	0	0	21.45357
Female	81	2/22/2007	2/22/2007	TT	GT	1	1	26.07897
Male	63		3/22/2007	CC	GT	1	0	22.02432
Male	78	4/18/2007	4/18/2007	TT	GT	0	1	28.40816
Female	74	3/29/2005	3/29/2005	CT	GT	1	1	26.06168
Female	68		4/18/2007	CT	GG	0	0	19.14063
Female	71		3/21/2007	CT	TT	0	0	21.79931
Female	76		4/19/2007	CT	GG	0	0	22.38631
Male	71		4/25/2007	CT	GT	1	0	23.49524
Female	67	3/15/2005	3/15/2005	CC	GT	1	1	23.62445
Female	80	4/5/2007	4/5/2007	CT	GG	0	0	23.72529
Male	77		3/28/2007	CT	GT	1	0	26.29849

Female	73	9/24/2004	9/24/2004	CC	GT	1	1	25.63117
Female	61		3/28/2007	TT	TT	0	0	30.46875
Male	78		3/29/2007	CT	GG	1	0	28
Male	71		6/11/2007	CC	TT	1	0	20.44674
Male	77		3/29/2007	CT	GT	1	0	23.93899
Male	75		4/18/2007	TT	GT	0	0	33.59375
Female	60	10/4/2005	10/4/2005	CC	GT	0	1	25.23634
Female	58		4/19/2007	CC	TT	1	0	25.12783
Female	72		4/5/2007	CC	GG	0	0	26.3958
Female	75	9/20/2005	9/20/2005	TT	GT	0	1	22.14533
Male	67		4/19/2007	CT	GT	1	0	26.66667
Female	82		4/25/2007	TT	GG	0	0	28.84153
Male	77		5/3/2007	TT	GT	1	0	25
Male	65	9/6/2005	9/6/2005	CT	GT	1	1	27.23922
Female	67		5/24/2007	CC	GG	1	0	22.65625
Male	76		5/3/2007	CT	GG	0	0	26.81359
Female	63	8/10/2006	8/10/2006	CC	GG	0	1	24.80159
Male	72		5/24/2007	CC	GG	1	0	22.86237
Male	81	5/24/2007	5/24/2007	CT	GG	1	1	20.91071
Female	78		6/7/2007	CT	TT	0	0	31.2213
Female	56		6/7/2007	CT	TT	0	0	20.77562
Male	75		6/7/2007	CT	GT	0	0	25.47666
Female	69		5/22/2007	CT	GT	0	0	23.87511
Male	77		6/21/2007	TT	GT	0	0	24.38653
Male	71		6/21/2007	CC	GT	1	0	27.43484
Female	78		8/16/2007	CC	GT	1	0	22.50693
Female	80		7/3/2007	CT	GT	0	0	28.73175
Female	80		6/4/2007	TT	TT	1	0	23.11111
Female	78		3/24/2005	CT	GT	1	0	22.53086
Female	75		6/7/2007	TT	GT	0	0	25.97012
Female	77	9/19/2006	9/19/2006	CT	GG	0	1	22.30815
Female	72	6/20/2006	6/20/2006	CT	GG	0	1	27.1809
Female	64		8/23/2007	CT	TT	0	0	19.14063
Male	60		8/23/2007	TT	GG	1	0	25.01352
Female	79		8/30/2007	TT	GG		0	24.78426
Female	82		9/6/2007	CT	TT	0	0	41.41519
Female	76		9/6/2007	CT	GG	1	0	29.96433
Female	72		9/6/2007	CT	GG	0	0	27.33564
Male	68		10/2/2007	CT	GT	1	0	25.5102
Female	77		10/4/2007	CC	GT	0	0	22.05805
Male	60		11/27/2007	CT	GG	0	0	28.73175
Female	73		10/4/2007	TT	GT	0	0	25
Male	72		10/15/2007	TT	GT	0	0	30.42185
Female	69		10/8/2007	CC	GT	0	0	24.81633
Female	74		10/8/2007	TT	GT	0	0	23.73866
Male	69		10/8/2007	CT	GT	1	0	19.53125
Female	75		11/5/2007	CT	TT	0	0	21.50189
Male	80		10/15/2007	CC	TT	1	0	23.5102
Female	56		9/27/2007	CT	GG	0	0	24.85907
Female	83		10/15/2007	CC	GT	0	0	25.60554
Female	79		4/21/2005	CC	TT	1	0	26.79494
Female	60		10/22/2007	CC	GG	0	0	22.49135

Female	69		10/15/2007	CC	TT	0	0	29.29688
Male	82	11/7/2006	11/7/2006	TT	TT	1	1	24.22145
Female	79	9/11/2006	9/11/2006	CT	GT	0	1	34.15978
Female	67		11/5/2007	TT	TT	0	0	35.55556
Female	65		11/5/2007	TT	GT	1	0	27.04164
Female	80		11/20/2007	CC	TT	0	1	24.80159
Male	76		11/27/2007	CC	GT	1	0	26.29758
Female	81	4/27/2005	4/27/2005	CC	GT	0	1	28
Female	75		11/27/2007	CC	GT	1	0	27.68878
Male	75		11/27/2007	CC	GT	1	0	24.38653
Female	73		11/5/2007	CT	GT	1	0	24.97399
Female	77	11/14/2005	11/14/2005	CT	GT	0	1	22.26563
Male	82	8/22/2007	8/22/2007	CT	GT	1	1	25.80645
Female	84		12/4/2007	TT	GT	0	0	22.59814
Female	80		12/4/2007	CT	GT	1	0	26.89767
Female	72		12/4/2007	CT	GG	0	0	24.4646
Female	83		6/7/2005	CT	GT	0	0	22.65625
Female	69	5/29/2007	5/29/2007	CT	GT	0	1	23.62445
Male	76		12/10/2007	CT	GG	0	0	24.22145
Female	79	10/3/2006	10/3/2006	CC	GG	0	1	24.44444
Female	70	3/8/2005	3/8/2005	CT	GT	1	1	20.3125
Female	65		12/10/2007	CC	GT	1	0	26.12245
Female	72		12/10/2007	CT	GG	1	0	24.44444
Female	82		12/10/2007	TT	TT	0	0	33.21799
Female	72		1/7/2008	CT	GT	0	0	23.30668
Male	79		12/10/2007	CT	GT	1	0	29.0688
Male	78		1/7/2008	CT	GT	1	0	34.96358
Female	63		1/17/2008	CT	GT	0	0	24.73246
Male	73		1/17/2008	CT	GT	1	0	23.52941
Male	75		1/21/2008	CT	TT	1	0	28.30385
Female	80		7/5/2005	CT	GG	0	0	26.72993
Female	76		1/21/2008	CT	GG	0	0	28.3737
Female	81	1/23/2007	1/23/2007	CT	GT	0	1	23.11111
Female	82		1/24/2008	CT	GT	0	0	27.68878
Female	71		1/25/2005	CC	TT	0	0	36.88889
Male	73		1/21/2008	CT	GT	1	0	24.7721
Female	68		1/17/2008	CC	TT	0	0	28.40816
Female	84	12/4/2007	12/4/2007	CC	GT	1	1	23.87511
Female	83		1/21/2008	TT	TT	0	0	23.42209
Male	63		1/17/2008	TT	GT	1	0	28.34186
Female	72	2/11/2008	2/11/2008	TT	TT	0	1	24.14152
Male	77		2/4/2008	CC	GT	1	0	27.5802
Female	71	6/29/2007	6/29/2007	CT	GG	1	1	25.34435
Female	55	4/28/2007	4/28/2007	CT	TT	0	1	27.40766
Female	76		2/4/2008	CC	GT	1	0	21.96712
Female	78	2/6/2007	2/6/2007	CT	GT	0	1	26.25958
Male	68		2/11/2008	CC	GT	1	0	23.50781
Female	76		1/28/2008	CC	GG	0	0	31.88776
Male	73		2/11/2008	CT	TT	1	0	23.23346
Male	82		2/11/2008	TT	GT	0	0	25.39022
Female	80		2/25/2008	CC	GG	0	0	34.8944
Female	71	9/14/2005	9/14/2005	CT	TT	0	1	28.44095

Female	73	1/28/2008	1/28/2008	CT	TT	0	1	20.44914
Female	77		2/11/2008	CC	GG	0	0	24.91349
Female	68		1/28/2008	CC	GT	1	0	27.77427
Male	66	11/20/2006	11/20/2006	CT	TT	1	1	24.30462
Female	76	5/15/2007	5/15/2007	CT	TT	0	1	22.60026
Female	68	1/3/2006	1/3/2006	CT	GT	1	1	25.72103
Male	80	11/17/2005	11/17/2005	CC	GG	1	1	24.62296
Male	73	10/18/2005	10/18/2005	CT	TT	0	1	24.00549
Female	71		3/10/2008	CT	GG	0	0	22.60026
Male	73		3/10/2008	CT	GT	1	0	26.63892
Female	77		2/25/2008	CC	GT	0	0	25.46939
Female	75	11/19/2007	11/19/2007	TT	GT	0	1	25.28011
Male	76		3/10/2008	TT	TT	0	0	32.28306
Female	78		3/31/2008	CT	GT	1	1	26.5625
Female	67		3/10/2008	CT	GG	0	0	21.96712
Female	74		3/31/2008	CT	GT	0	0	23.55556
Female	79		3/10/2008	CC	GG	0	0	27.20961
Female	75		3/31/2008	TT	GT	0	0	23.52941
Female	83		5/19/2008	CT	GG	0	0	26.07897
Female	76	2/10/2006	2/10/2006	CC	GG	1	1	19.69267
Male	70		3/31/2008	CC	TT	1	0	31.57207
Male	56		5/5/2008	CT	GG	0	0	24.21875
Female	82	8/17/2005	8/17/2005	TT	GT	0	1	22.14533
Female	76	9/6/2005	9/6/2005	TT	GT	0	1	21.7502
Female	84		4/14/2008	CT	GT	0	0	26.25958
Female	69	3/9/2007	3/9/2007	TT	GT	0	1	35.32124
Male	74		5/26/2008	CT	GT	1	0	25.23634
Female	77		4/14/2008	CC	GG	0	0	20.93212
Male	83		4/14/2008	CT	GT	1	0	24.91349
Female	72		3/31/2008	CC	GG	0	0	24.80159
Female	78		4/26/2005	CC	TT	0	0	19.72104
Male	83		4/14/2008	CC	GT	1	0	22.58955
Male	76	3/6/2007	3/6/2007	CC	GG	1	1	24.09297
Female	67		4/14/2008	CT	TT	0	0	26.37024
Male	81	2/10/2006	2/10/2006	CC	GG	1	1	21.21832
Male	78	5/19/2008	5/19/2008	CC	GT	1	1	22.26563
Female	62		5/19/2008	CT	GG	1	0	23.12467
Male	72		5/26/2005	CT	GT	1	0	24.9989
Female	76		5/19/2008	CC	GT	1	0	25.03992
Female	68		5/5/2008	TT	GT	0	0	25.23634
Female	72		5/19/2008	CT	TT	0	0	26.21882
Male	63		5/26/2008	TT	TT	0	0	25.71166
Male	77		5/26/2008	CT	TT	1	0	
Female	74		5/26/2008	CT	TT	1	0	26.17188
Male	77		5/26/2008	CC	GG	0	0	27.74475
Female	79		6/9/2008	TT	GG	1	0	25.71166
Female	56		6/9/2008	CC	GT	0	0	21.77755
Male	66		6/9/2008	TT	GG	0	0	30.11099
Female	78	4/3/2006	4/3/2006	CT	TT	0	1	27.4406
Male	84		6/16/2005	CC	GT	1	0	16.52893
Female	74	6/20/2006	6/20/2006	CT	GT	0	1	18.66667
Female	58		6/2/2008	CT	GT	0	0	29.75779

Female	75	9/7/2006	9/7/2006	CT	GT	1	1	23.50781
Male	76		6/2/2008	CC	GT	1	0	27.14304
Male	77		6/2/2008	CT	GT	1	0	32.03125
Male	67	11/17/2005	11/17/2005	CC	GG	1	1	24.4898
Female	77		6/9/2008	CT	TT	0	0	23.66144
Male	76		6/23/2008	CC	GT	1	0	36.1231
Female	71	2/26/2008	2/26/2008	CT	GT	0	1	24.24392
Male	79		6/23/2008	CT	GG	1	0	24.69136
Female	81	5/14/2007	5/14/2007	TT	TT	0	1	32.87311
Female	74		6/23/2008	CT	GG	0	0	25.34435
Male	72		6/23/2008	CC	GT	1	0	23.04688
Male	79		9/1/2008	CC	GG	1	0	27.73438
Female	77		8/25/2008	CT	GG	0	0	25.10957
Female	78		8/25/2008	TT	TT	0	0	25.45807
Female	78		9/22/2008	CT	GG	0	0	27.46914
Female	65	2/16/2005	2/16/2005	CT	GT	0	1	25.71166
Female	71		9/1/2008	TT	TT	0	0	25.88757
Female	58		8/25/2008	TT	GT	1	0	37.46098
Female	73		9/1/2008	CT	TT	1	0	23.82813
Male	70		9/15/2008	CT	GT	1	0	21.09619
Female	80	2/12/2007	2/12/2007	CT	GT	0	1	21.60494
Male	72	3/10/2006	3/10/2006	CT	GG	0	1	25.05931
Male	76		9/29/2008	TT	GG	1	0	22.03857
Female	59		9/15/2008	CC	GT	0	0	24.85795
Female	58		9/8/2008	CC	GT	0	0	24.60938
Female	82		9/15/2008	TT	GG	0	0	31.11111
Male	80		9/8/2008	CT	GT	1	0	27.08416
Male	73		9/8/2008	CT	GG	1	0	18.98659
Male	70		9/15/2008	TT	GT	1	0	22.86237
Male	72		9/8/2008	CT	TT	0	1	26.12861
Male	84		9/1/2008	TT	GT	0	0	30.11028
Female	67		9/15/2008	TT	TT	0	0	21.48438
Female	56	9/26/2006	9/26/2006	CC	GT	0	1	24.16716
Male	66		9/22/2008	CC	TT	0	0	21.87242
Male	74		9/29/2008	CC	TT	1	0	25.88057
Female	74	1/6/2006	1/6/2006	CT	GT	0	1	22.58271
Female	84	9/15/2008	9/15/2008	TT	GT	0	1	25.52964
Male	85	10/3/2006	10/3/2006	CC	GT	0	1	25
Female	77		9/29/2008	CT	GG	0	0	29.6875
Female	73	3/17/2006	3/17/2006	TT	GG	0	1	21.96712
Female	76		9/29/2008	CT	GT	0	0	29.27099
Male	70		9/22/2008	CT	TT	1	0	23.03005
Female	62		9/29/2008	CT	TT	1	0	
